# Difference in anterior tibial subluxation measured with the bone axis method predicts high‐grade pivot shift in ACL‐deficient knees: A multicenter cohort study

**DOI:** 10.1002/jeo2.70675

**Published:** 2026-02-27

**Authors:** Nobuaki Hayashi, Shotaro Watanabe, Tsuyoshi Hamada, Manato Horii, Masahiko Saito, Yuta Muramatsu, Yusuke Sato, Taisuke Fukawa, Ryuichiro Akagi, Ryosuke Nakagawa, Seiji Kimura, Satoshi Yamaguchi, Seiji Ohtori, Takahisa Sasho

**Affiliations:** ^1^ Department of Orthopaedic Surgery, Graduate School of Medical and Pharmaceutical Sciences Chiba University Chiba Japan; ^2^ Department of Orthopaedic Surgery, Center for Preventive Medical Sciences Chiba University Chiba Japan; ^3^ Orthopaedic Surgery Chiba Medical Center Chiba Japan; ^4^ Orthopaedic Surgery Kitachiba Spine & Sports Clinic Chiba Japan; ^5^ Orthopaedic Surgery Eastern Chiba Medical Center Chiba Japan; ^6^ Orthopaedic Surgery Japanese Red Cross Narita Hospital Chiba Japan; ^7^ Orthopaedic Surgery Oyumino Central Hospital Chiba Japan; ^8^ Orthopaedic Surgery Kohnodai Hospital Chiba Japan; ^9^ Graduate School of Global and Transdisciplinary Studies Chiba University Chiba Japan

**Keywords:** anterior cruciate ligament, anterior tibial subluxation, magnetic resonance imaging, pivot shift test, rotational knee instability

## Abstract

**Purpose:**

To determine which magnetic resonance imaging (MRI)‐based anterior tibial subluxation (ATS) measurement best reflects rotational instability under anaesthesia in anterior cruciate ligament (ACL)‐injured patients.

**Methods:**

This retrospective multicenter cohort study included 291 patients who underwent ACL reconstruction between October 2022 and December 2024. Preoperative MRI measurements of lateral ATS (L‐ATS) and difference between lateral and medial ATS (D‐ATS) were obtained using the plateau method (PM) and the bone axis method (BAM). Rotational instability was assessed under anaesthesia using the pivot shift test and categorized as low‐grade (LG‐PS) or high‐grade (HG‐PS). Receiver operating characteristic (ROC) curve analysis was performed to evaluate the discriminative ability of each measurement. Using the cutoff value derived from the Youden index of the ATS parameter that demonstrated the largest ROC‐AUC, multivariate logistic regression identified independent predictors of HG‐PS.

**Results:**

Compared with the LG‐PS group, D‐ATS measured with PM (*p* < 0.001) and both L‐ATS and D‐ATS measured with BAM (*p* = 0.035 and *p* < 0.001, respectively) were greater in the HG‐PS group. D‐ATS measured with BAM demonstrated the highest ROC‐AUC (0.675) among measurement methods, with a cutoff value of 3.8 mm. In multivariate analysis, D‐ATS measured with BAM ≥ 3.8 mm (odds ratio [OR]: 3.92, *p* < 0.001), female sex (OR: 1.88, *p* = 0.025), contralateral knee hyperextension (OR: 3.70, *p* < 0.001) and medial meniscal injury (OR: 2.27, *p* = 0.004) were independent predictors of HG‐PS.

**Conclusions:**

Among MRI‐based methods for measuring ATS in ACL‐injured knees, D‐ATS measured with BAM best reflected pivot shift grade under anaesthesia and was an independent predictor of HG‐PS. A D‐ATS measured with BAM value ≥ 3.8 mm may help identify patients at risk of rotational instability and, in combination with other clinical factors, assist preoperative surgical planning.

**Level of Evidence:**

Level III, cohort study.

**Abbreviations**
ACL: anterior cruciate ligament
ACLR: anterior cruciate ligament reconstruction
ATS: anterior tibial subluxation
ATS‐BAM: anterior tibial subluxation measured with the bone axis method
ATS‐PM: anterior tibial subluxation measured with the plateau method
AUC: area under the curve
BAM: bone axis method
BMI: body mass index
CI: confidence interval
D‐ATS: difference between lateral and medial anterior tibial subluxation
HG‐PS: high‐grade pivot shift
ICC: intraclass correlation coefficient
LG‐PS: low‐grade pivot shift
L‐ATS: lateral anterior tibial subluxation
MRI: magnetic resonance imaging
OR: odds ratio
PCL: posterior cruciate ligament
PM: plateau method
PTS: posterior tibial slope
ROC: receiver operating characteristic
TAS: Tegner activity scale

## INTRODUCTION

Anterior cruciate ligament (ACL) injuries result in both anterior and rotational instability of the knee [16]. Manual tests commonly used to evaluate knee instability include the Lachman test for anterior instability and the pivot shift test for rotational instability. A preoperative high‐grade pivot shift test under anaesthesia has been associated with an increased risk of ACL graft failure after reconstruction [10], and with residual rotational instability following ACL reconstruction (ACLR) [17, 18, 34].

Accurate assessment of rotational instability in awake patients is often limited owing to muscular guarding [3]. Lateral compartment translation and tibial acceleration during the pivot shift test are significantly smaller in awake patients compared with those under anaesthesia [4, 24]. Without an accurate evaluation of rotational instability until anaesthesia is administered on the day of surgery, appropriate surgical planning, graft selection and risk assessment may be less informed by the true magnitude of rotational laxity. Although these decisions are routinely made preoperatively using clinical examination, imaging findings and patient‐related factors, the ability to predict the pivot shift grade under anaesthesia could further refine surgical decision‐making, including optimal graft choice [14, 25, 30] and consideration of additional lateral extra‐articular procedures [6, 7, 8, 9, 31].

Since anterior tibial subluxation (ATS) was first reported to be associated with failed ACL reconstruction and knee instability [1, 5], numerous subsequent studies have been published. Magnetic resonance imaging (MRI) allows objective quantification of ATS, and two primary measurement methods have been described: the plateau method (PM), which uses a line perpendicular to the tibial articular surface, and the bone axis method (BAM), which references the longitudinal tibial axis. ATS can be measured separately in the medial and lateral compartments, and lateral ATS (L‐ATS) has been reported to correlate with pivot shift test grade under anaesthesia [2, 22, 23, 35]. Additionally, the difference between lateral and medial ATS (D‐ATS) has also been reported to correlate with the pivot shift test grade under anaesthesia [28, 37]. Notably, ATS measurements demonstrate satisfactory inter‐ and intra‐observer reliability [37, 38].

ATS can be assessed using L‐ATS or D‐ATS, each measured using either PM or BAM. However, it remains unclear which measurement method most accurately reflects rotational instability of the knee. Therefore, the primary aim of this study was to determine which MRI‐based ATS parameter best discriminates high‐grade pivot shift (HG‐PS) under anaesthesia in ACL‐injured patients. The secondary aim was to investigate whether the ATS parameter with the highest discriminatory ability is an independent predictor of HG‐PS after adjustment for clinically relevant covariates. It was hypothesized that the ATS parameter that best reflects pivot shift grade under anaesthesia would remain an independent predictor of HG‐PS.

## MATERIALS AND METHODS

### Study design

This retrospective multicenter cohort study utilized prospectively collected data from an ACL reconstruction database.

### Patient selection

Patients who underwent ACL reconstruction at the participating facilities between October 2022 and December 2024 were included. All patients underwent manual physical examination and MRI before surgery, and ACL ruptures were confirmed arthroscopically. Clinical and imaging evaluations were performed by board‐certified orthopaedic surgeons specializing in knee surgery, each with more than 10 years of clinical experience.

Exclusion criteria were as follows: associated fractures; age <10 years or >60 years; revision ACLR; previous surgery on the affected or contralateral knee; combined ligament injuries (posterior cruciate ligament [PCL] and/or Grade 3 collateral ligament injuries) and radiographic evidence of osteoarthritis (Kellgren–Lawrence grade ≥2). Patients with Grade 1 or 2 collateral ligament injuries were not excluded. Meniscal injuries were not considered exclusion criteria. Additional exclusion criteria were: time from MRI to surgery exceeding 6 months; knee flexion angle <0° or >30° at the time of MRI; and non‐qualifying MRI images, defined as examinations performed on scanners with field strength <1.5 T or with imaging coverage insufficient to define the bone axis.

### Data collection and variables

All participating institutions used a standardized registration form to record demographic data, including age, sex, and body mass index (BMI), Tegner Activity Scale (TAS) score [33], comorbidities, surgical history, cause and date of injury, range of motion, presence of contralateral knee hyperextension, Lachman test grade [11], pivot shift test grade [11], date of surgery and associated intra‐articular injuries including meniscal and cartilage lesions. All preoperative MRI images were acquired in the clinical setting as part of standard care and subsequently registered in the study database. Registration in the database was considered complete once all the required data were collected.

### Evaluation of knee instability

Knee range of motion and instability were assessed under anaesthesia. Contralateral knee hyperextension was defined as >10°. The Lachman and pivot shift tests were performed on the injured knee and graded according to the International Knee Documentation Committee classification.

The Lachman test was performed with the knee in 25° of flexion, placing one hand medially on the tibia and the other laterally on the femur. Grades were defined as follows: Grade A, normal anterior translation; Grade B, nearly normal translation; Grade C, abnormal translation and Grade D, severely abnormal translation [11]. In this study, Grades A and B were considered low‐grade instability, and Grades C and D as high‐grade instability.

The pivot shift test was performed by holding the heel, applying internal rotation and valgus stress to the tibia and flexing the knee from full extension. Grading was as follows: Grade A, negative pivot shift (normal knee); Grade B, nearly normal pivot shift with a glide (+); Grade C, abnormal pivot shift with a clunk (++) and Grade D, severely abnormal pivot shift with a gross shift (+++) [11]. As with the Lachman test, Grades A and B were categorized as low‐grade instability and Grades C and D as high‐grade instability.

Across institutions, grading criteria and operational definitions for the pivot shift tests were aligned through inter‑institutional consensus meetings to promote consistency of performance and interpretation. In total, pivot shift testing and grading were performed by eight board‐certified knee surgeons across the participating institutions.

### Evaluation of intra‐articular injuries

ACLR was performed under general or spinal anaesthesia. Intra‐articular assessment was conducted via anterolateral and anteromedial portals using a probe. ACL tears were confirmed arthroscopically. The medial and lateral menisci were probed to evaluate the size and type of tears, and findings were recorded. Meniscal injuries requiring repair or resection were defined as medial meniscal tears, ramp lesions, lateral meniscal tears and lateral meniscal posterior horn tears.

### MRI measurement methods

Knee MRI scans obtained from the participating institutions were used for analysis. Sagittal proton density–weighted sequences were analysed. All measurements were performed by a single board‐certified orthopaedic surgeon specializing in knee surgery. Images were analysed using AZE VirtualPlace Plus (Canon Medical Systems Corporation).

**Figure 1:**
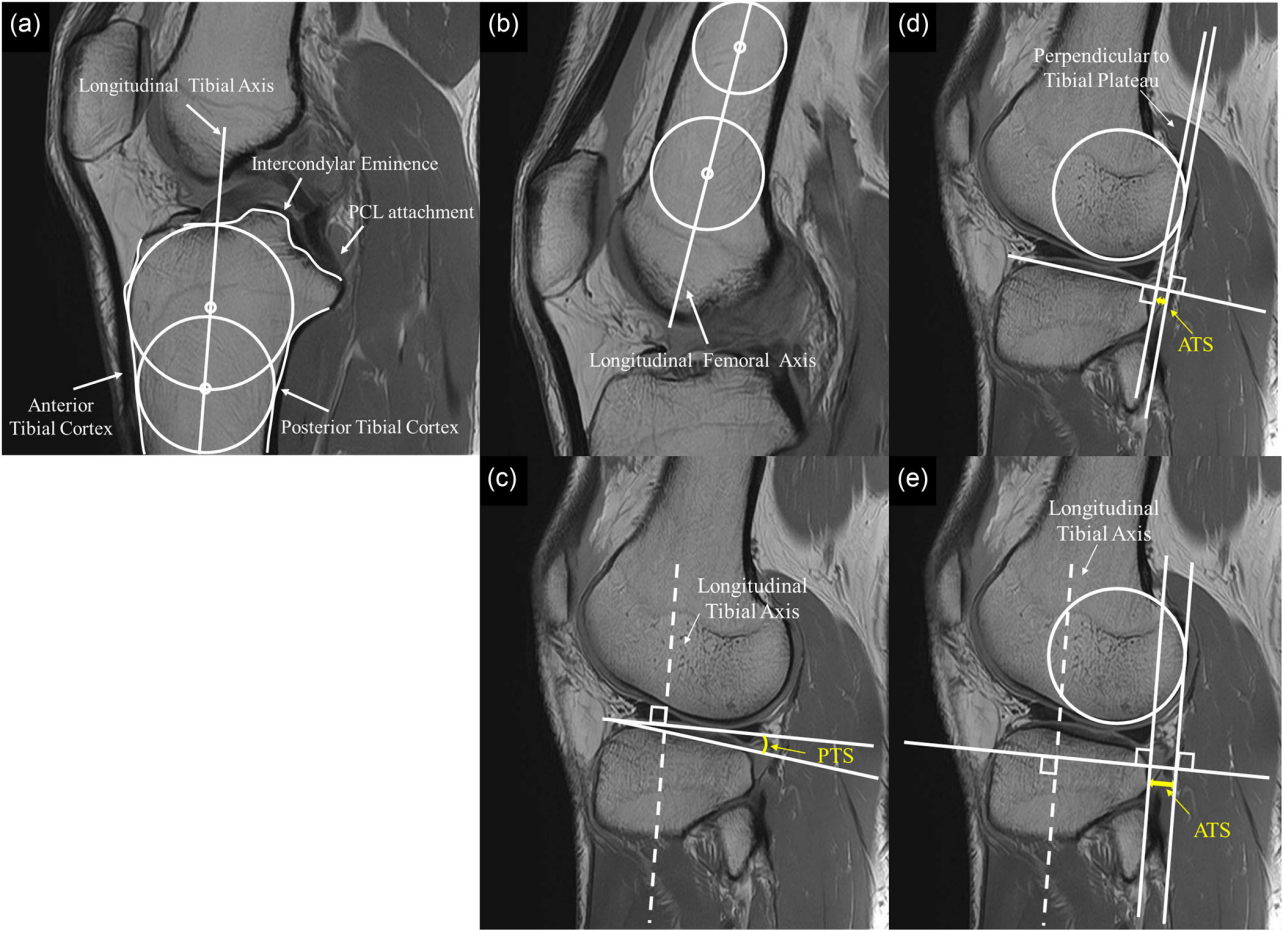
Measurements of knee angle, ATS and PTS on sagittal magnetic resonance imaging. (a) To define the longitudinal tibial axis, two circles were drawn. The tibial axis was defined as the line connecting the centres of these two circles. (b) To define the longitudinal femoral axis, two circles were drawn. The femoral axis was defined as the line connecting the centres of these two circles. (c) PTS was defined as the angle between the tibial plateau and a line perpendicular to the longitudinal tibial axis. (d) ATS measured with the plateau method was defined as the distance between a vertical line of tibial plateau drawn from the posterior margin to the posterior femoral condyle and the posterior edge of the tibial articular surface. (e) ATS measured with the bone axis method was defined as the distance between a parallel line of the tibial axis drawn from the posterior margin to the posterior femoral condyle and a parallel line drawn from the posterior margin of the tibial articular surface. ATS, anterior tibial subluxation; PCL, posterior cruciate ligament; PTS, posterior tibial slope.

The longitudinal tibial axis was assessed on the central sagittal slice, where the tibial intercondylar eminence, anterior tibial cortex and concave posterior tibial cortex were all visible. To define the longitudinal tibial axis, two circles were drawn: the proximal circle was fitted to the anterior, posterior and proximal cortical borders; the distal circle was positioned such that its centre laid on the circumference of the proximal circle and touched the anterior and posterior cortices. The longitudinal tibial axis was defined as the line connecting the centres of these two circles (Figure 1a) [32]. The longitudinal femoral axis was assessed on the sagittal slice most clearly showing the femoral shaft. Two circles were drawn, each fitted to the anterior and posterior cortical borders of the femoral shaft, and the longitudinal femoral axis was defined as the line connecting their centres (Figure 1b) [36].

The knee flexion angle was measured as the angle between the femoral and tibial axes. The medial sagittal slice was defined as the slice where the medial head of the gastrocnemius tendon inserts into the femur. The lateral slice was defined as the slice at the medial‐most aspect of the proximal tibiofibular joint. Using these slices, ATS and posterior tibial slope (PTS) were measured.

The joint surface line was defined as the line connecting the anterior and posterior edges of the cartilage‐covered tibial plateau. PTS was measured as the angle between the tibial plateau and a line perpendicular to the tibial axis, representing the posterior inclination of the articular surface (Figure 1c) [15, 30].

ATS measured with PM (ATS‐PM) was defined as the distance between a vertical line from the posterior margin of a circle fitted to the posterior femoral condyle and a vertical line from the posterior edge of the tibial articular surface [15, 32]. ATS measured with BAM (ATS‐BAM) was defined as the distance between a line parallel to the tibial axis drawn from the posterior margin of the circle fitted to the posterior femoral condyle and a parallel line from the posterior margin of the tibial articular surface [37]. Anterior tibial translation was defined as positive (Figure 1d,e).

MRI measurements included knee flexion angle, lateral PTS, and L‐ATS and D‐ATS measured using both PM and BAM. The intraclass correlation coefficient (ICC) was calculated to assess intra‐ and inter‐observer reliability for L‐ATS and D‐ATS measured with both the plateau and BAMs in 20 cases. These cases were independently measured by a second board‐certified orthopaedic surgeon, and the first examiner repeated all measurements after an interval of more than 1 month.

### Statistical analysis

Demographic characteristics and clinical features (age, sex, BMI, mechanism of injury, TAS, time from injury to surgery, contralateral knee hyperextension and Lachman grade) were compared between the low‐grade pivot shift (LG‐PS) and HG‐PS groups. Continuous variables were presented as mean ± standard deviation or median with interquartile range, as appropriate. Categorical variables were presented as number and percentage. Continuous variables were analysed using either the Student's *t* test or the Wilcoxon rank‐sum test, depending on data normality. Categorical variables were analysed using the *χ* ^2^ test.

Reliability of intra‐observer and inter‐observer measurements was assessed using the ICC, which ranges from 0 (no agreement) to 1 (perfect agreement) [19]. Specifically, ICC (1,2) was used for intra‐observer reliability and ICC (2,1) for inter‐observer reliability. In addition, the standard error of measurement was calculated to quantify absolute measurement error and is reported alongside ICC values.

For the primary analysis, Student's *t* tests compared L‐ATS and D‐ATS measured with both PM and BAM between LG‐PS and HG‐PS groups. Receiver operating characteristic (ROC) curves were generated using HG‐PS as the positive condition, and the area under the curve (AUC) was calculated to assess discriminatory ability. The optimal cutoff value for each parameter was determined using the Youden index (sensitivity − [1 − specificity]). Sensitivity and specificity at the optimal cutoff were calculated.

Using the ATS parameter that demonstrated the largest ROC‐AUC, multivariate logistic regression was performed to determine whether ATS remained a significant predictor of pivot shift grade after adjustment for other factors. Pivot shift grade was entered as the dependent variable, and age, sex, BMI, time from injury to surgery, knee hyperextension, medial meniscus tears, lateral meniscus tears, knee angle on MRI and ATS were categorized into high and low groups according to the cutoff value were included as independent variables.

As a secondary analysis, the relationship between ATS and PTS was examined to assess whether PM underestimates ATS compared with BAM due to the influence of PTS. Pearson correlation coefficients [21] were calculated between L‐ATS‐BAM and lateral PTS; L‐ATS‐PM and lateral PTS and the difference in L‐ATS between the two methods and lateral PTS.

All statistical analyses were conducted using JMP®, Version 18 (SAS Institute Inc., 1989–2025), with statistical significance set at *p* < 0.05.

## RESULTS

### Study participants and group allocation

**Figure 2:**
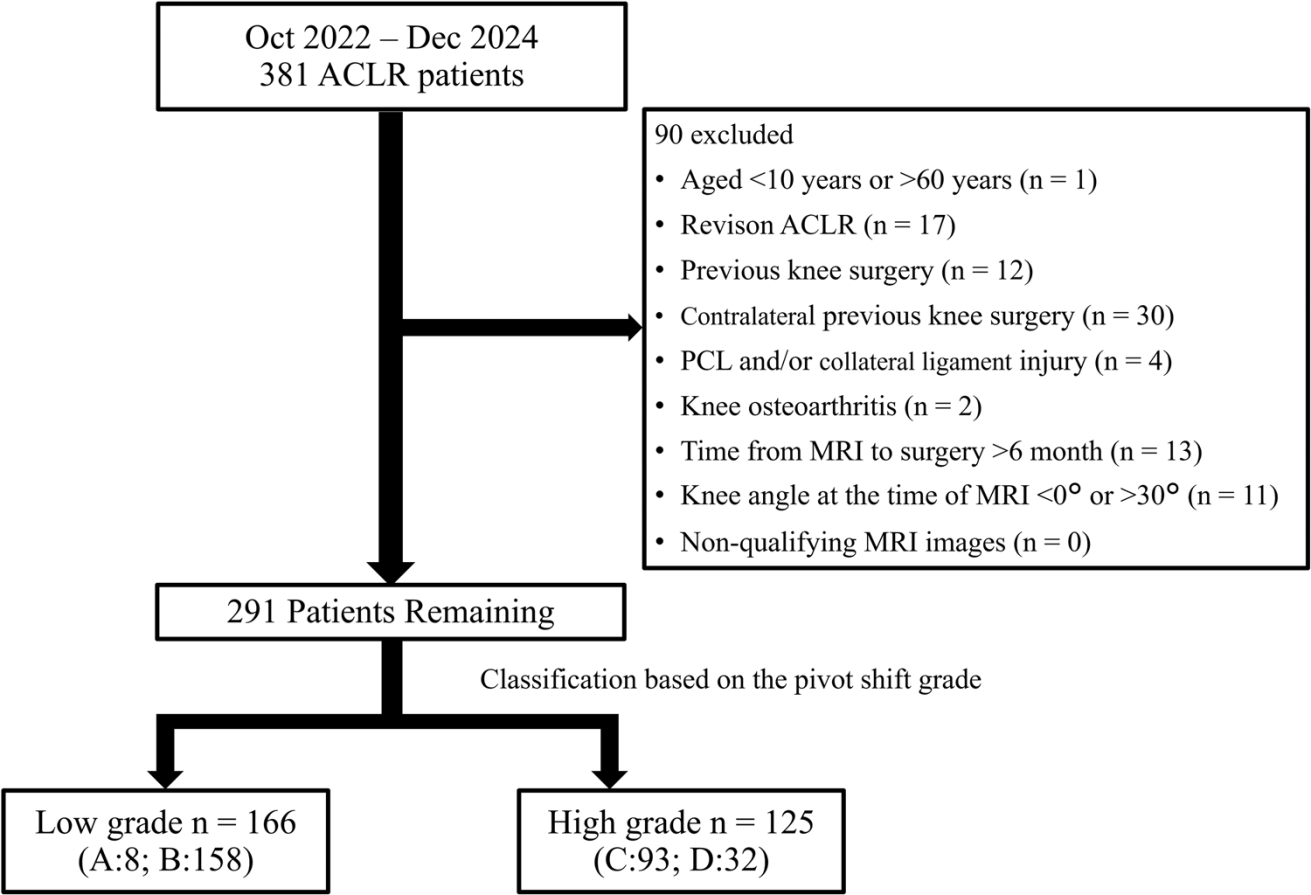
Flowchart of study participants. After excluding ineligible cases from the initial 381 knees, patients were classified into two groups based on the pivot shift test. ACLR, anterior cruciate ligament reconstruction; MRI, magnetic resonance imaging; PCL, posterior cruciate ligament.

A total of 381 patients underwent ACLR, of whom 90 were excluded. Ultimately, 291 patients were included in the final statistical analysis. Classification based on the pivot shift test under anaesthesia revealed 8 patients (2.7%) with Grade A, 158 (54.3%) with Grade B, 93 (32.0%) with Grade C and 32 (11.0%) with Grade D. Accordingly, 166 patients (57.0%) were allocated to the LG‐PS group and 125 patients (43.0%) to the HG‐PS group (Figure 2).

### Comparison of patient characteristics

Patient demographics and clinical characteristics are summarized in Table 1. There were no significant differences between the LG‐PS and HG‐PS groups with respect to age, sex, BMI, mechanism of injury, TAS score, time from injury to surgery or lateral meniscus tears. In contrast, significant differences were observed in the incidence of contralateral knee hyperextension (*p* < 0.001), Lachman test grade (*p* = 0.017), medial meniscal tears (*p* = 0.001), ramp lesions (*p* = 0.005) and lateral meniscal posterior horn tears (*p* = 0.023).

**Table 1:** Patient characteristics for all cases and by group.^a^

	All cases (*n* = 291)	LG‐PS (*n* = 166)	HG‐PS (*n* = 125)	*p* value
Age (years)	28.4 ± 12.7	28.8 ± 12.4	27.9 ± 13.2	0.22
Sex				0.19
Male	158 (54.3%)	96 (57.8%)	62 (49.6%)	
Female	133 (45.7%)	70 (42.2%)	63 (50.4%)	
Body mass index, kg/m^2^	23.8 ± 3.9	23.5 ± 3.6	24.1 ± 4.2	0.17
Mechanism of injury				0.95
Contact	67 (23.0%)	38 (22.9%)	29 (23.2%)	
Noncontact	224 (77.0%)	128 (77.1%)	96 (76.8%)	
Tegner Activity Scale	6 (6–7)	6 (6–7)	6 (6–7)	0.71
Time to surgery, months^b^	2 (1–5)	2 (1–5)	3 (1–6)	0.14
Knee hyperextension	48 (16.5%)	13 (7.8%)	35 (28.0%)	**<0.001**
Lachman test^c^				**0.017**
Low grade (A or B)	190 (65.3%)	118 (71.1%)	72 (57.6%)	
High grade (C or D)	101 (34.7%)	48 (28.9%)	53 (42.4%)	
Meniscal tear				
Medial meniscus	102 (35.1%)	45 (27.1%)	57 (45.6%)	**0.001**
Ramp lesion	49 (16.8%)	19 (11.5%)	30 (24.0%)	**0.005**
Lateral meniscus	147 (50.5%)	79 (47.6%)	68 (54.4%)	0.25
Lateral meniscus posterior horn	21 (7.2%)	7 (4.2%)	14 (11.2%)	**0.023**

*Note*: Bold numbers indicate a significant difference (<0.05).

^a^ Data are presented as mean ± standard deviation, number (*n*) or percentage (%) and median with interquartile range.

^b^ Time to surgery was treated as a continuous variable, measured in months.

^c^ The Lachman test was classified as low grade for Grades A and B and high for Grades C and D.

### Reliability of intra‐ and inter‐observer measurements

Intra‐observer reliability was excellent for both L‐ATS and D‐ATS measured with PM and BAM. Inter‐observer reliability was also excellent for L‐ATS measured by both methods and good for D‐ATS measured by both methods (Table 2).

**Table 2:** Intra‐ and inter‐observer reliability for ATS.

	Intraclass correlation coefficient (standard error of measurement [mm])			
	L‐ATS		D‐ATS	
	Plateau method	Bone axis method	Plateau method	Bone axis method
Intra‐observer	0.954 (1.41)	0.965 (1.07)	0.963 (0.95)	0.946 (1.26)
	Excellent	Excellent	Excellent	Excellent
Inter‐observer	0.918 (1.34)	0.943 (1.00)	0.830 (1.40)	0.870 (1.23)
	Excellent	Excellent	Good	Good

Abbreviations: D‐ATS, difference between lateral and medial anterior tibial subluxation; L‐ATS, lateral anterior tibial subluxation.

### ATS measurements on MRI

When comparing the LG‐PS and HG‐PS groups, no significant difference was observed in L‐ATS‐PM (*p* = 0.44). However, D‐ATS‐PM (*p* < 0.001) and both L‐ATS and D‐ATS measured with BAM (*p* = 0.035 and *p* < 0.001, respectively) were significantly greater in the HG‐PS group. No significant difference in the knee flexion angle was noted between the two groups. Lateral PTS was significantly greater in the HG‐PS group (Table 3).

**Table 3:** Results of magnetic resonance imaging measurements.^a^

	All cases (*n* = 291)	LG‐PS (*n* = 166)	HG‐PS (*n* = 125)	*p* value
Knee angle, degree	12.0 ± 6.4	11.8 ± 6.2	12.2 ± 6.7	0.64
Posterior tibial slope, degree	5.8 ± 3.0	5.5 ± 2.9	6.2 ± 3.2	**0.041**
Plateau method, mm				
L‐ATS	2.40 ± 3.78	2.25 ± 3.75	2.60 ± 3.82	0.44
D‐ATS	1.70 ± 3.23	1.12 ± 3.04	2.46 ± 3.33	**<0.001**
Bone axis method, mm				
L‐ATS	5.70 ± 4.34	5.24 ± 4.23	6.32 ± 4.42	**0.035**
D‐ATS	2.97 ± 3.56	2.00 ± 3.22	4.25 ± 3.58	**<0.001**

*Note*: Bold numbers indicate a significant difference (*p* < 0.05).

Abbreviations: D‐ATS, difference between lateral and medial anterior tibial subluxation; HG‐PS, high‐grade pivot shift test; L‐ATS, lateral anterior tibial subluxation; LG‐PS, low‐grade pivot shift test.

^a^ Data are presented as mean ± standard deviation.

**Figure 3:**
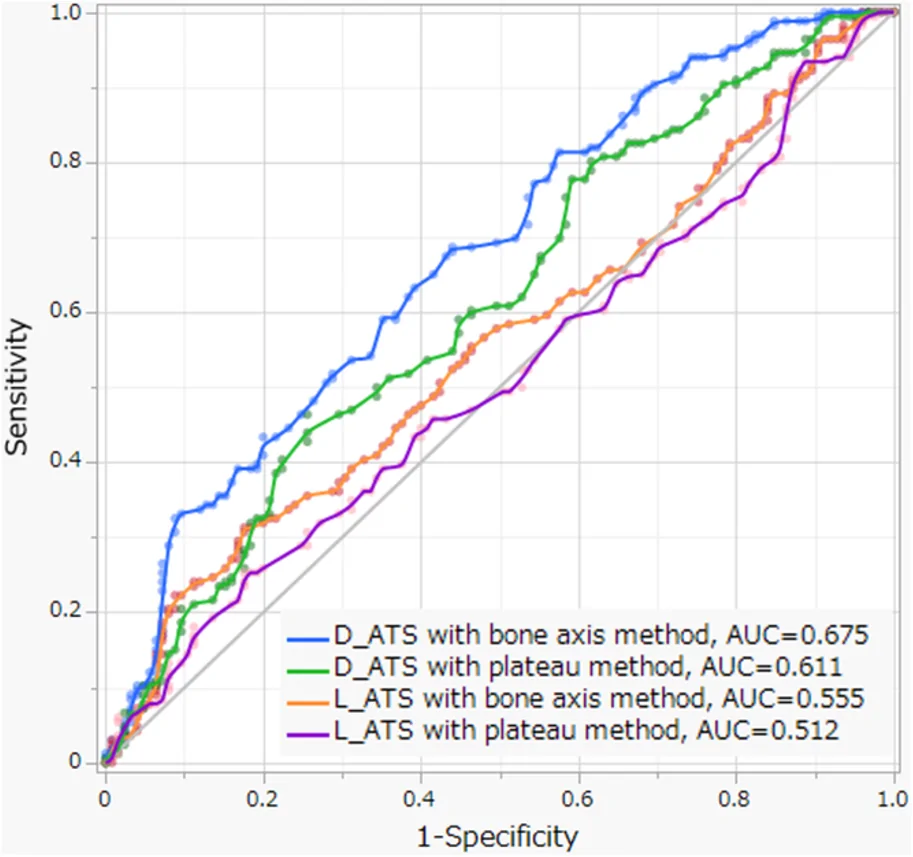
Receiver operating characteristic curves of L‐ATS and D‐ATS measured with the plateau and bone axis methods for high‐grade pivot shift. AUC, area under the receiver operating characteristic curve; D‐ATS, difference between lateral and medial anterior tibial subluxation; L‐ATS, lateral anterior tibial subluxation.

ROC‐AUC values were L‐ATS‐PM, 0.512; D‐ATS‐PM, 0.611; L‐ATS‐BAM, 0.555 and D‐ATS‐BAM, 0.675 (Table 4, Figure 3).

**Table 4:** Diagnostic performance of ATS for high‐grade pivot shift.

	ROC‐AUC	Cutoff value (mm)	Youden Index	Sensitivity	Specificity
Plateau method					
L‐ATS	0.512	–0.4	0.077	0.824	0.253
D‐ATS	0.611	1.0	0.208	0.744	0.464
Bone axis method					
L‐ATS	0.555	3.0	0.137	0.824	0.313
D‐ATS	0.675	3.8	0.247	0.560	0.687

Abbreviations: ATS, anterior tibial subluxation; D‐ATS, difference between lateral and medial anterior tibial subluxation; L‐ATS, lateral anterior tibial subluxation; ROC‐AUC, area under the receiver operating characteristic curve.

The cutoff values and corresponding Youden indices were L‐ATS‐PM, –0.4 mm (0.077); D‐ATS‐PM, 1.0 mm (0.208); L‐ATS‐BAM, 3.0 mm (0.137) and D‐ATS‐BAM, 3.8 mm (0.247).

### Multivariate analysis with high‐grade pivot shift as the dependent variable

Among the ATS parameters, D‐ATS‐BAM showed the largest ROC‐AUC, with the cutoff value of 3.8 mm. Therefore, patients were dichotomized into two groups based on D‐ATS‐BAM of 3.8 mm, and this variable was included as one of the explanatory factors in the multivariate logistic regression analysis. Significant predictors of HG‐PS were female sex (odds ratio [OR]: 1.88, 95% confidence interval [CI]: 1.08–3.27, *p* = 0.025), contralateral knee hyperextension (OR: 3.70, 95% CI: 1.74–7.89, *p* < 0.001), medial meniscal tears (OR: 2.27, 95% CI: 1.31–3.93, *p* = 0.004) and high D‐ATS‐BAM (OR: 3.92, 95% CI: 2.24–6.84, *p* < 0.001) (Table 5).

**Table 5:** Multivariate logistic regression analysis for high‐grade pivot shift.^a^

	High‐grade pivot shift	
Variables	OR (95% CI)	*p* value
Age	0.99 (0.97–1.01)	0.22
Sex		
Male	1.00 (Reference)	
Female	1.88 (1.08–3.27)	**0.025**
Body mass index	1.07 (1.00–1.15)	0.058
Time to surgery^b^	1.00 (1.00–1.01)	0.22
Knee hyperextension	3.70 (1.74–7.89)	**<0.001**
Medial meniscus tear	2.27 (1.31–3.93)	**0.004**
Lateral meniscus tear	1.11 (0.66–1.88)	0.69
Knee angle	1.01 (0.97–1.06)	0.57
High D‐ATS with bone axis method^c^	3.92 (2.24–6.84)	**<0.001**

*Note*: Bold numbers indicate a significant difference (*p* < 0.05).

Abbreviations: CI, confidence interval; D‐ATS, difference between lateral and medial anterior tibial subluxation; OR, odds ratio.

^a^ Odds ratios were adjusted for all other predictors in the table.

^b^ Time to surgery was treated as a continuous variable, measured in months.

^c^ High D‐ATS with bone axis method was defined as ≥3.8 mm.

### Correlation between ATS and PTS

**Figure 4:**
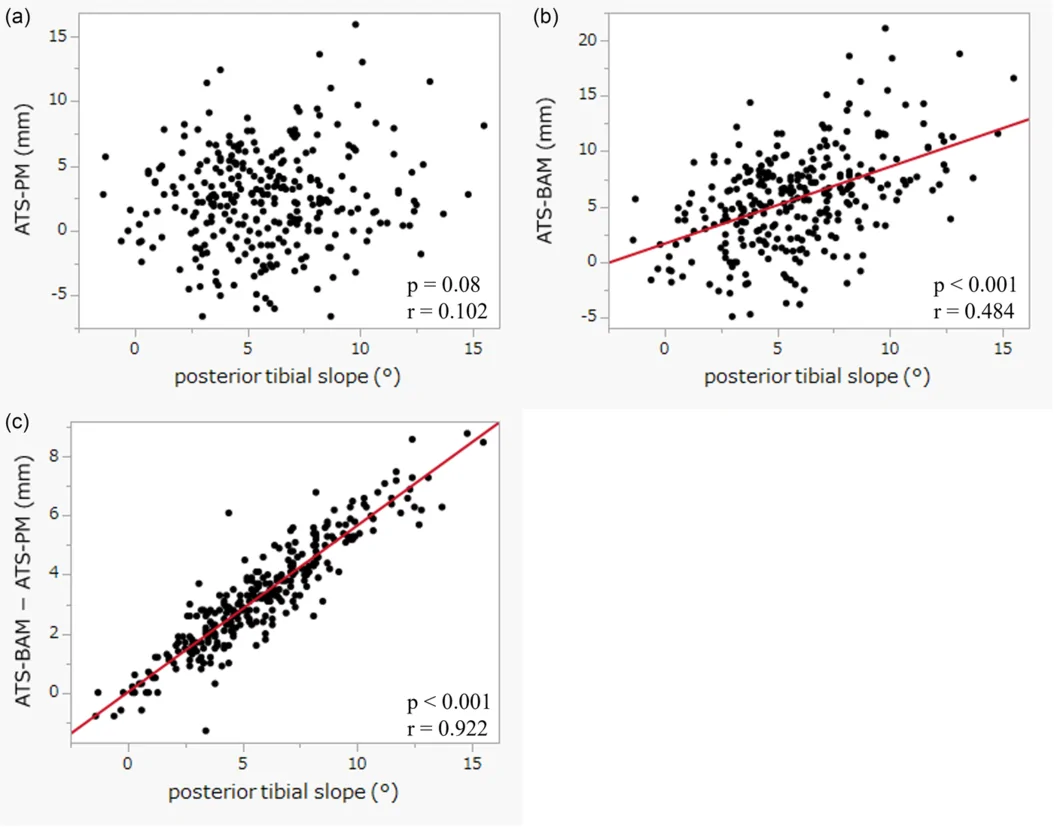
Correlation between lateral ATS and lateral PTS. (a) Correlation between lateral ATS measured with the plateau method and lateral PTS. (b) Correlation between lateral ATS measured with the bone axis method and lateral PTS. (c) Correlation between the difference in lateral ATS values measured with the bone axis and plateau methods, and lateral PTS. ATS‐BAM, anterior tibial subluxation measured with the bone axis method; ATS‐PM, anterior tibial subluxation measured with the plateau method; PTS, posterior tibial slope.

No significant correlation was observed between L‐ATS‐PM and lateral PTS (*r* = 0.102, *p* = 0.081). In contrast, L‐ATS‐BAM demonstrated a moderate correlation with lateral PTS (*r* = 0.484, *p* < 0.001). Furthermore, the difference between L‐ATS measured with BAM and PM showed an extremely strong correlation with PTS (*r* = 0.922, *p* < 0.001) (Figure 4).

## DISCUSSION

In this study, preoperative MRI‐based ATS was associated with the pivot shift grade under anaesthesia in patients undergoing ACLR. D‐ATS‐BAM provided the best discrimination for HG‐PS (ROC‐AUC 0.675), and a cutoff of ≥3.8 mm independently predicted HG‐PS.

Several previous studies have reported that L‐ATS‐PM is associated with rotational instability [2, 22, 23, 35]. More recently, based on the concept that D‐ATS may reflect tibial internal rotation, an association between D‐ATS and rotational instability has also been reported [28, 38]. In a retrospective study analysing 156 knees using weight‐bearing MRI, patients with D‐ATS‐PM > 6.0 mm had a significantly higher incidence of HG‐PS compared to those with D‐ATS < 3.0 mm. Multivariate analysis in that study identified D‐ATS and lateral meniscal posterior horn tears as significant predictors of HG‐PS [28]. Another retrospective study comparing 104 ACL‐injured and 104 uninjured patients reported significantly greater L‐ATS and D‐ATS values, both measured with BAM, in the ACL‐injured group [38]. Furthermore, in a study investigating the relationship between D‐ATS‐BAM and anterolateral complex injury, the mean D‐ATS‐BAM among ACL‐injured patients with anterolateral complex injury was 5.6 ± 3.2 mm [39]. In this cohort, D‐ATS had a higher ROC‐AUC than L‐ATS when measured with either method, suggesting that D‐ATS may better reflect rotational instability than L‐ATS. Multivariate analysis confirmed that D‐ATS‐BAM ≥ 3.8 mm was a significant predictor of HG‐PS, even when controlling for other covariates. Female sex, contralateral knee hyperextension and medial meniscal injury were also found to be independent predictors, and D‐ATS‐BAM ≥ 3.8 mm showed the largest odds ratio among the explanatory variables. These findings suggest that patients with high D‐ATS‐BAM ≥ 3.8 mm, as well as those with these clinical risk factors, may require careful consideration regarding surgical strategy.

Although most previous studies on MRI‐based assessment of ATS have evaluated ATS‐PM, several recent reports have described ATS‐BAM [37, 38]. The methodological difference between PM and BAM has been attributed to the influence of tibial morphology on PM, whereas BAM more directly reflects the femorotibial relationship [37]. Consistent with this concept, the difference between L‐ATS‐BAM and L‐ATS‐PM increased with PTS, supporting the susceptibility of PM measurements to individual tibial geometry. This may partly explain why D‐ATS‐BAM outperformed D‐ATS‐PM in terms of ROC‐AUC, as did L‐ATS‐BAM compared with L‐ATS‐PM. Although MRI does not capture the full length of the tibia and therefore yields a tibial axis different from that obtained on full‐length radiographs [13], the method used in this study demonstrated good reliability, as it allows for accurate measurement even on standard knee imaging.

Preoperative assessment of rotational instability under anaesthesia is considered valuable for surgical planning. The pivot shift test performed in awake patients may not reliably reflect actual instability owing to muscular guarding [3]. Without a reliable assessment until the day of surgery, preoperative planning, including graft selection and risk assessment, may remain insufficient. MRI is widely performed in patients prior to ACLR and is a highly accessible imaging modality. Therefore, D‐ATS‐BAM on routinely obtained MRI may help predict rotational instability under anaesthesia and contribute to more informed surgical decision‐making, including appropriate graft choice [14, 25, 30] and whether to perform lateral extra‐articular procedures [6, 7, 8, 9, 31]. Furthermore, D‐ATS‐BAM may also be useful for assessing residual rotational instability after ACLR in situations where clinical evaluation is limited by muscular guarding.

## LIMITATIONS

There are some limitations to this study. First, the pivot shift test is a subjective assessment and may vary among examiners [3, 12, 20, 27]. In this multicenter study, pivot shift testing and grading were performed by eight surgeons, and inter‐observer variability should be considered an important limitation. To minimize inter‐observer variability, the testing procedures and grading criteria were standardized across participating institutions, and consistency in evaluation was confirmed. In addition, dichotomizing the four‐grade pivot shift into low‐ and high‐grade groups may oversimplify the dynamic nature of rotational instability. Second, knee flexion angles during MRI acquisition varied among patients. As flexion angle may influence ATS measurements [26, 29], we excluded cases with substantial deviation to minimize its impact. Third, the time interval from MRI to surgery was not uniform, which could have affected instability due to injury progression. To address this, cases with more than 6 months interval between MRI and surgery were excluded. Fourth, patients with concomitant Grade 1 or 2 collateral ligament injuries were not excluded, which may have influenced pivot shift findings and potentially affected the observed associations. Fifth, the discriminative ability of D‐ATS‐BAM was modest (AUC 0.675), and because the Youden index was modest, the proposed cutoff value may be useful for research stratification but should be applied with caution in clinical decision‐making. Finally, MRI is a static modality and cannot capture dynamic aspects of instability. Because rotational laxity and the pivot shift phenomenon are multifactorial, HG‐PS cannot be explained by MRI‐based ATS alone.

## CONCLUSION

Among various methods for measuring ATS on preoperative MRI in ACL‐injured knees, D‐ATS‐BAM showed the strongest association with the pivot shift test under anaesthesia. In multivariate analysis, D‐ATS‐BAM ≥ 3.8 mm was independently associated with a high‐grade pivot shift. These findings suggest that D‐ATS‐BAM may be used as an adjunct MRI marker to complement clinical assessment and, in combination with other clinical and imaging factors, help guide surgical strategy.

## AUTHOR CONTRIBUTIONS

*Conception and design*: Nobuaki Hayashi, Shotaro Watanabe and Takahisa Sasho. *Analysis and interpretation of data*: Nobuaki Hayashi, Shotaro Watanabe and Takahisa Sasho. *Acquisition of data*: All authors. *Drafting of the article*: Nobuaki Hayashi and Shotaro Watanabe. *Critical revision of the article for important intellectual content and final approval*: All authors. *Accuracy of the work*: All authors. *Funding acquisition*: Nobuaki Hayashi and Shotaro Watanabe. All authors had full access to all data in the study and take responsibility for the integrity of the data and the accuracy of the data analysis.

## CONFLICT OF INTEREST STATEMENT

Seiji Ohtori has received lecture fees and honoraria from Daiichi Sankyo Co., Ltd., Nippon Zoki Pharmaceutical Co., Ltd. and Hisamitsu Pharmaceutical Co., Inc. The remaining authors declare no conflict of interest.

## ETHICS STATEMENT

The study protocol was approved by the Ethics Committee of Chiba University Hospital (Approval No. M10346) and the ethics committees of all participating institutions. Written informed consent was obtained from all patients prior to surgery.

## Data Availability

Data supporting the findings of this study are available from the corresponding author upon reasonable request.

## References

[1] Almekinders L , Chiavetta J , Clarke J . Radiographic evaluation of anterior cruciate ligament graft failure with special reference to tibial tunnel placement. Arthroscopy. 1998;14(2):206–211. 10.1016/s0749-8063(98)70042-8 9531134

[2] Bedi A , Musahl V , Lane C , Citak M , Warren RF , Pearle AD . Lateral compartment translation predicts the grade of pivot shift: a cadaveric and clinical analysis. Knee Surg Sports Traumatol Arthrosc. 2010;18(9):1269–1276. 10.1007/s00167-010-1160-y 20480356

[3] Benjaminse A , Gokeler A , van der Schans CP . Clinical diagnosis of an anterior cruciate ligament rupture: a meta‐analysis. J Orthop Sports Phys Ther. 2006;36(5):267–288. 10.2519/jospt.2006.2011 16715828

[4] Caracciolo G , Yáñez R , Silvestre R , De la Fuente C , Zamorano H , Ossio A , et al. Intraoperative pivot‐shift accelerometry combined with anesthesia improves the measure of rotatory knee instability in anterior cruciate ligament injury. J Exp Orthop. 2021;8(1):80. 10.1186/s40634-021-00396-1 PMC8463650 34561730

[5] Dejour H , Bonnin M . Tibial translation after anterior cruciate ligament rupture. Two radiological tests compared. J Bone Joint Surg Br. 1994;76(5):745–749. 8083263

[6] Firth AD , Bryant DM , Litchfield R , McCormack RG , Heard M , MacDonald PB , et al. Predictors of graft failure in young active patients undergoing hamstring autograft anterior cruciate ligament reconstruction with or without a lateral extra‐articular tenodesis: the stability experience. Am J Sports Med. 2022;50(2):384–395. 10.1177/03635465211061150 PMC8829733 35050817

[7] Geeslin AG , Moatshe G , Chahla J , Kruckeberg BM , Muckenhirn KJ , Dornan GJ , et al. Anterolateral knee extra‐articular stabilizers: a robotic study comparing anterolateral ligament reconstruction and modified Lemaire lateral extra‐articular tenodesis. Am J Sports Med. 2018;46(3):607–616. 10.1177/0363546517745268 29268024

[8] Getgood AMJ , Bryant DM , Litchfield R , Heard M , McCormack RG , Rezansoff A , et al. Lateral extra‐articular tenodesis reduces failure of hamstring tendon autograft anterior cruciate ligament reconstruction: 2‐year outcomes from the STABILITY study randomized clinical trial. Am J Sports Med. 2020;48(2):285–297. 10.1177/0363546519896333 31940222

[9] Green DW , Hidalgo Perea S , Brusalis CM , Chipman DE , Asaro LA , Cordasco FA . A modified Lemaire lateral extra‐articular tenodesis in high‐risk adolescents undergoing anterior cruciate ligament reconstruction with quadriceps tendon autograft: 2‐year clinical outcomes. Am J Sports Med. 2023;51(6):1441–1446. 10.1177/03635465231160681 36917840

[10] Gupta R , Kapoor A , Singhal A , Patil BM , Bansal P . The presence of high‐grade pivot shift test preoperatively is associated with inferior functional outcomes. Phys Sportsmed. 2022;50(4):306–310. 10.1080/00913847.2021.1924047 33910466

[11] Hefti F , Müller W , Jakob RP , Stäubli HU . Evaluation of knee ligament injuries with the IKDC form. Knee Surg Sports Traumatol Arthrosc. 1993;1(3–4):226–234. 10.1007/BF01560215 8536037

[12] Hoshino Y , Araujo P , Ahlden M , Moore CG , Kuroda R , Zaffagnini S , et al. Standardized pivot shift test improves measurement accuracy. Knee Surg Sports Traumatol Arthrosc. 2012;20(4):732–736. 10.1007/s00167-011-1850-0 22205096

[13] Hudek R , Schmutz S , Regenfelder F , Fuchs B , Koch PP . Novel measurement technique of the tibial slope on conventional MRI. Clin Orthop Relat Res. 2009;467(8):2066–2072. 10.1007/s11999-009-0711-3 PMC2706341 19190973

[14] Itoh M , Itou J , Okazaki K , Iwasaki K . Estimation failure risk by 0.5‐mm differences in autologous hamstring graft diameter in anterior cruciate ligament reconstruction: a meta‐analysis. Am J Sports Med. 2024;52(2):535–543. 10.1177/03635465221150654 36876736

[15] Iwaki H , Pinskerova V , Freeman MAR . Tibiofemoral movement 1: the shapes and relative movements of the femur and tibia in the unloaded cadaver knee. J Bone Joint Surg Br. 2000;82(8):1189–1195. 10.1302/0301-620x.82b8.10717 11132285

[16] Kaeding CC , Léger‐St‐Jean B , Magnussen RA . Epidemiology and diagnosis of anterior cruciate ligament injuries. Clin Sports Med. 2017;36(1):1–8. 10.1016/j.csm.2016.08.001 27871652

[17] Kamada K , Matsushita T , Nagai K , Hoshino Y , Araki D , Kanzaki N , et al. Risk factors of residual pivot‐shift after anatomic double‐bundle anterior cruciate ligament reconstruction. Arch Orthop Trauma Surg. 2023;143(2):977–985. 10.1007/s00402-022-04428-y 35364734

[18] Kawanishi Y , Nozaki M , Kobayashi M , Yasuma S , Fukushima H , Murase A , et al. Preoperative knee instability affects residual instability as evaluated by quantitative pivot‐shift measurements during double‐bundle ACL reconstruction. Orthop J Sports Med. 2020;8(10):2325967120959020. 10.1177/2325967120959020 PMC7592323 33178876

[19] Koo TK , Li MY . A guideline of selecting and reporting intraclass correlation coefficients for reliability research. J Chiropr Med. 2016;15(2):155–163. 10.1016/j.jcm.2016.02.012 PMC4913118 27330520

[20] Kuroda R , Hoshino Y , Kubo S , Araki D , Oka S , Nagamune K , et al. Similarities and differences of diagnostic manual tests for anterior cruciate ligament insufficiency: a global survey and kinematics assessment. Am J Sports Med. 2012;40(1):91–99. 10.1177/0363546511423634 21989128

[21] Lachenbruch PA , Cohen J . Statistical power analysis for the behavioral sciences (2nd ed.). J Am Stat Assoc. 1989;84(408):1096.

[22] Lian J , Novaretti JV , Sheean AJ , Patel NK , Whaley S , Popchak A , et al. Static lateral tibial plateau subluxation predicts high‐grade rotatory knee laxity in anterior cruciate ligament‐deficient knees. Am J Sports Med. 2019;47(2):277–284. 10.1177/0363546518812435 30525899

[23] Liu A , Cui W , Yang W , Li C , Yan S , Xin Z , et al. Anterior tibial subluxation of lateral compartment is associated with high‐grade rotatory instability for acute but not chronic anterior cruciate ligament injuries: an magnetic resonance imaging case‐control study. Arthroscopy. 2022;38(10):2852–2860. 10.1016/j.arthro.2022.04.012 35550417

[24] Lopomo N , Signorelli C , Rahnemai‐Azar AA , Raggi F , Hoshino Y , Samuelsson K , et al.; PIVOT Study Group . Analysis of the influence of anaesthesia on the clinical and quantitative assessment of the pivot shift: a multicenter international study. Knee Surg Sports Traumatol Arthrosc. 2017;25(10):3004–3011. 10.1007/s00167-016-4130-1 27095250

[25] Mariscalco MW , Flanigan DC , Mitchell J , Pedroza AD , Jones MH , Andrish JT , et al. The influence of hamstring autograft size on patient‐reported outcomes and risk of revision after anterior cruciate ligament reconstruction: a Multicenter Orthopaedic Outcomes Network (MOON) Cohort Study. Arthroscopy. 2013;29(12):1948–1953. 10.1016/j.arthro.2013.08.025 PMC3844091 24140144

[26] Morishige Y , Harato K , Oki S , Kaneda K , Niki Y , Nakamura M , et al. Four‐dimensional computed tomographic analysis of screw home movement in patients with anterior cruciate ligament deficient knee—a 3D‐3D registration technique. Skeletal Radiol. 2022;51(8):1679–1685. 10.1007/s00256-021-03986-3 35006277

[27] Musahl V , Hoshino Y , Ahlden M , Araujo P , Irrgang JJ , Zaffagnini S , et al. The pivot shift: a global user guide. Knee Surg Sports Traumatol Arthrosc. 2012;20(4):724–731. 10.1007/s00167-011-1859-4 22210541

[28] Ni Q‐K , Wang X‐P , Guo Q , Li M , Liu N , Zhang H . High‐grade pivot‐shift phenomenon after anterior cruciate ligament injury is associated with asymmetry of lateral and medial compartment anterior tibial translation and lateral meniscus posterior horn tears. Knee Surg Sports Traumatol Arthrosc. 2022;30(11):3700–3707. 10.1007/s00167-022-06972-x 35460039

[29] Noh JH , Nam WD , Roh YH . Anterior tibial displacement on preoperative stress radiography of ACL‐injured knee depending on knee flexion angle. Knee Surg Relat Res. 2019;31(1):14. 10.1186/s43019-019-0014-2 PMC7219608 32660620

[30] Snaebjörnsson T , Hamrin Senorski E , Ayeni OR , Alentorn‐Geli E , Krupic F , Norberg F , et al. Graft diameter as a predictor for revision anterior cruciate ligament reconstruction and KOOS and EQ‐5D values: a cohort study from the Swedish National Knee Ligament Register based on 2240 patients. Am J Sports Med. 2017;45(9):2092–2097. 10.1177/0363546517704177 28460194

[31] Sonnery‐Cottet B , Saithna A , Cavalier M , Kajetanek C , Temponi EF , Daggett M , et al. Anterolateral ligament reconstruction is associated with significantly reduced ACL graft rupture rates at a minimum follow‐up of 2 years: a prospective comparative study of 502 patients from the SANTI Study Group. Am J Sports Med. 2017;45(7):1547–1557. 10.1177/0363546516686057 28151693

[32] Tanaka MJ , Jones KJ , Gargiulo AM , Delos D , Wickiewicz TL , Potter HG , et al. Passive anterior tibial subluxation in anterior cruciate ligament‐deficient knees. Am J Sports Med. 2013;41(10):2347–2352. 10.1177/0363546513498995 23928320

[33] Tegner Y , Lysholm J . Rating systems in the evaluation of knee ligament injuries. Clin Orthop Relat Res. 1985;198(198):42–49. 4028566

[34] Yamamoto Y , Tsuda E , Maeda S , Naraoka T , Kimura Y , Chiba D , et al. Greater laxity in the anterior cruciate ligament‐injured knee carries a higher risk of postreconstruction pivot shift: intraoperative measurements with a navigation system. Am J Sports Med. 2018;46(12):2859–2864. 10.1177/0363546518793854 30193083

[35] Ye Z , Wu X , Chen J , Cho E , Xie G , Dong S , et al. Association between anterior tibial subluxation of lateral compartment and high‐grade knee laxity in patients with anterior cruciate ligament deficiency. Am J Sports Med. 2023;51(7):1698–1707. 10.1177/03635465231166712 37092733

[36] Yoshihara A , Siboni R , Nakagawa Y , Mouton C , Jacquet C , Nakamura T , et al. Lateral‐medial asymmetry of posterior tibial slope and small lateral tibial plateau articular surface depth are morphological factors of lateral meniscus posterior root tears in ACL‐injured patients. Knee Surg Sports Traumatol Arthrosc. 2023;31(9):3594–3603. 10.1007/s00167-023-07317-y 36656347

[37] Zhang Z , Pan X , Maimaitijiang P , Meng L , He Z , Zhao Q , et al. Anterior tibial subluxation measured under a modified protocol is positively correlated with posterior tibial slope: a comparative study of MRI measurement methods. Knee Surg Sports Traumatol Arthrosc. 2022;30(10):3350–3360. 10.1007/s00167-022-06913-8 35218376

[38] Zhang Z , Wang C , Maimaitimin M , Huang H , Pan X , Maimaitijiang P , et al. Anterior and rotational tibial subluxation in the setting of anterior cruciate ligament injuries: an MRI analysis. Knee. 2021;33:365–373. 10.1016/j.knee.2021.10.012 34753026

[39] Zhang Z , Yin Y , Bai W , Shi W , Pan X , Huang H , et al. Association of concomitant MRI‐determined anterolateral complex injury with quantitative measurements of altered rotational tibiofemoral position on MRI in patients with ACL injury. Orthop J Sports Med. 2024;12(2):23259671241230950. 10.1177/23259671241230954 PMC10898314 38414665

